# Effects of a person-centred and health-promoting intervention in home care services– a non-randomized controlled trial

**DOI:** 10.1186/s12877-021-02661-5

**Published:** 2021-12-18

**Authors:** Kristina Lämås, Karin Bölenius, Per-Olof Sandman, Marie Lindkvist, David Edvardsson

**Affiliations:** 1 Department of Nursing, Umeå University, 90187 Umeå, Sweden; 2 Department of Statistics, Umeå School of Business, Economics and Statistics (USBE), Umeå University, Umeå, Sweden; 3 Division of Epidemiology and Global Health, Department of Public Health and Clinical Medicine, Umeå University, Umeå, Sweden; 4 School of Nursing and Midwifery, La Trobe University, Melbourne, Australia

**Keywords:** Home care service, Intervention, Older adults, Person-centred care

## Abstract

**Background:**

Home care recipients have reported little self-determination and opportunity to influence their own care. Person-centred care focusing on involvement has improved the quality of life of older adults in health care and nursing homes; however, knowledge about the effects of person-centred interventions in aged care at home is sparse. The aim of this study was to study the effects of a person-centred and health-promoting intervention, compared with usual care, on health-related quality of life, thriving and self-determination among older adults, and on job satisfaction, stress of conscience and level of person-centred care among care staff.

**Methods:**

This is a non-randomized controlled trial with a before/after design. Participants from five home care districts in one municipality in northern Sweden were recruited to an intervention or control group. We evaluated health-related quality of life, thriving and self-determination among older home care recipients, and job satisfaction, person-centred care and stress of conscience among care staff. Evaluation was performed by questionnaires and responses were analysed using parametric and non-parametric statistical analyses.

**Results:**

Eighty-one older adults and 48 staff were included in the study. A clinically moderate and statistically significant difference between the intervention and control groups was found in thriving and negative emotions among older adults. The intervention contributed to maintaining high thriving levels, in contrast to decreased thriving in the control group (intervention: + 1, control: − 4, p 0.026, CI: − 10. 766, − 0.717). However, the intervention group rated an increase in negative emotions, while the control group was unchanged (intervention: − 7 control: + − 0, p 0.048, CI: − 17.435, − 0.098). No significant effects were found among staff.

**Conclusions:**

The intervention contributed to maintaining high levels of thriving in contrast to low levels found in the control group, and it seems reasonable to consider the intervention focus on staff as more person-centred and health-promoting. The finding that the intervention group had increase in negative emotions is difficult to interpret, and warrants further exploration. Even though the results are sparse, the challenges discussed may be of importance for future studies in the context of HCS.

**Trial registration:**

NCT02846246. Date of registration: 27 July 2016.

## Background

In many parts of the Western world an increasing percentage of older adults with declining health are offered care at home [1–5]. A reason for this is the growing population aged 65 years and above, so that in several Western countries the number of nursing home beds per 1000 population has declined in recent years [3]. In addition to the fact that people live longer, developments in society have been to create possibilities for older adults to live at home for as long as possible.

Remaining at home despite the need for care has been found to be preferred to moving to a nursing home [6]. One reason why older people wish to remain at home has been found to be linked to the positive experience of independence and autonomy when living at home [5]. Preserving autonomy is in turn an important factor for the experience of mental health and wellbeing [7].

A complicating factor in relation to home care service (HCS) is that HCS recipients have been reported to have little self-determination and opportunity to influence their own care in having their needs met [8]. A cross-sectional study in Sweden [9] found that higher self-determination among HCS recipients was associated with higher health-related quality of life (HRQoL). The opportunity to influence own care is therefore suggested to be an important factor when striving for high-quality HCS.

In the Scandinavian countries, “thriving” is a frequently used, everyday word. It is used to describe an experience of enjoying to be in a specific place or environment. Haight et al. [10] posit that the experience of thriving results from a well-adjusted interaction between the person and their human and non-human environment. However, thriving as a concept in care for older people has been scantily studied. In a cross-sectional study of HCS, thriving has been found to be associated with self-determination and taking part in social activities [11]. In nursing homes, thriving has been found to be associated with social activities [12]. Unfortunately, support for social activities has been reported to have low priority in HCSs [13]. In a cross-sectional study among people with dementia living at home in the UK, the top three unmet needs were related to social activities: daytime activities, company, and psychological distress [14]. Similarly, in a cross-sectional study in Sweden among people receiving HCSs, only 17% received help related to social needs [15]. It has been described that home care (HC) recipients’ social needs are given little attention in allocation of care resources as the need assessment process largely focuses on physical care-related needs [13].

In recent decades, the need for a person-centred approach in health care has been stressed [16, 17]. Person-centred care (PCC) has been defined in several, similar ways, for example by McCormack & McCance [18] who describe it as –.

*… an approach to practice established through the formation and fostering of therapeutic relationships between all care providers, older people and others significant to them in their lives. It is underpinned by values of respect for persons, individual right to self-determination, mutual respect and understanding. It is enabled by cultures of empowerment that foster continuous approaches to practice development. [18 p. 13]*

In research about institutional care in a nursing and a multidisciplinary context, PCC has been reported to be associated with increased quality of life (QoL) [19] and increased satisfaction with care in older adults [20]. Person-centred care has also been described to have positive effects on staff. From the point of view of staff, the provision of PCC is associated with increased job satisfaction [21, 22] and lower levels of job strain and stress of conscience among staff [23]. A systematic review by Blake et al. [24] found that seven out of eight interventions were effective at increasing PCC behaviour among health care staff working with people with dementia.

There are few studies that have focused on PCC delivered at home by different professionals [25–27], and even fewer focusing on PCC delivered by staff in the HCS. In the context of HCSs, interventions intended to increase PCC have been reported but often in the context of development projects [e.g. [28]]; as far as we know, they have not been research-driven.

In summary, there is some evidence that PCC could affect QoL and thriving among care recipients. Since the model of PCC emphasizes shared decision making it is also reasonable to assume that the experience of self-determination could be affected. Moreover, PCC is suggested to have a positive effect on job satisfaction, stress of conscience, and level of PCC among staff. However, the interventions and outcome measures have differed between studies, which is why it seems important to conduct further, controlled research about the effects of PCC interventions. Overall, the evidence is still sparse, especially in the HCS context, and further exploration seems to be needed. The aim of this study was to study the effects of a person-centred and health-promoting intervention, compared with usual care, on HRQoL, thriving and self-determination among older adults, and on job satisfaction, stress of conscience and level of PCC among care staff.

## Methods

### Design and setting of the study

This study was a non-randomized controlled trial with a before/after design. The study was conducted in 2016–2018 in a municipality in northern Sweden. Home care services in Sweden are largely publicly financed by taxes, and this includes the HCSs in this study. The care needs are assessed by a care assessor who, based on the assessment, decides on the level of HCS needed [13]. The HCS includes personal care and household work. There is less focus on social support, a shortcoming that has been suggested to be related to strained municipal economy which has led to further rationalizations in recent years [29]. Swedish HCSs have been criticized for having become increasingly limited, standardized and fragmented because of limited financial resources [29].

### Participants

All HCS districts in the municipality were invited to participate in the study. As described in our study protocol [30], a power calculation based on HRQoL as the primary outcome (measured using the Nottingham Health Profile (NHP)) showed that 270 HCS recipients needed to be included to reach a power of 85% at the 0.05 significance level. Five HCS districts were included based on the HCS managers’ reported interest in participating. The HCS districts were pragmatically allocated to either the intervention group or the control group by researchers; the aim was to have a comparable number of HCS recipients in both groups.

**Fig. 1:**
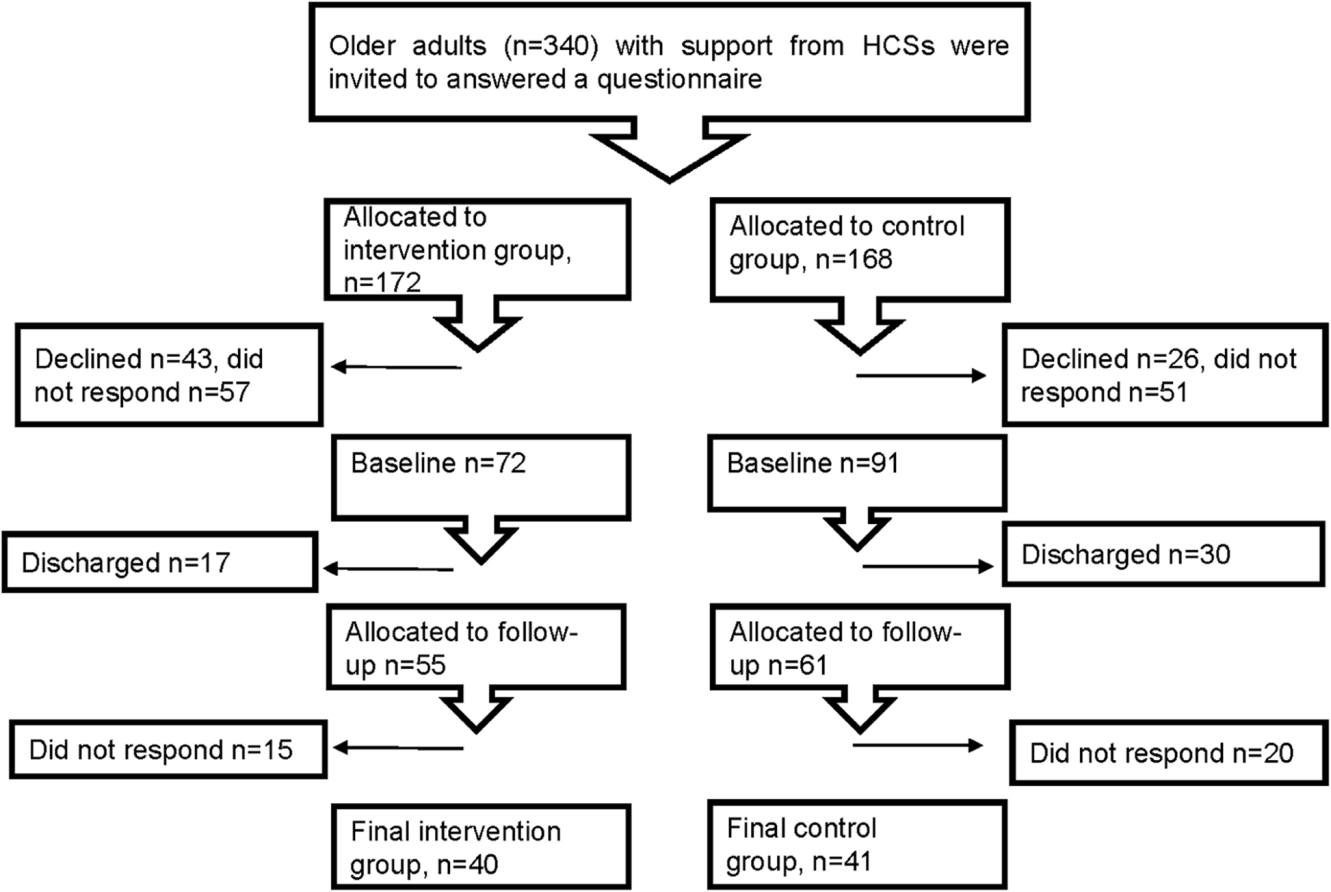
Flow chart of home care service (HCS) recipients participating in the study

Inclusion criteria for HCS recipients were: age 65 years or older, living at home with assistance from the HCS, and speaking and understanding Swedish. Exclusion criteria were: suffering from any condition that impedes communication. Of 340 invited HCS recipients, 163 (48%) agreed to participate, and 81 (24%) answered the questionnaire at both baseline and follow-up and were included in the study (Fig. 1).

**Fig. 2:**
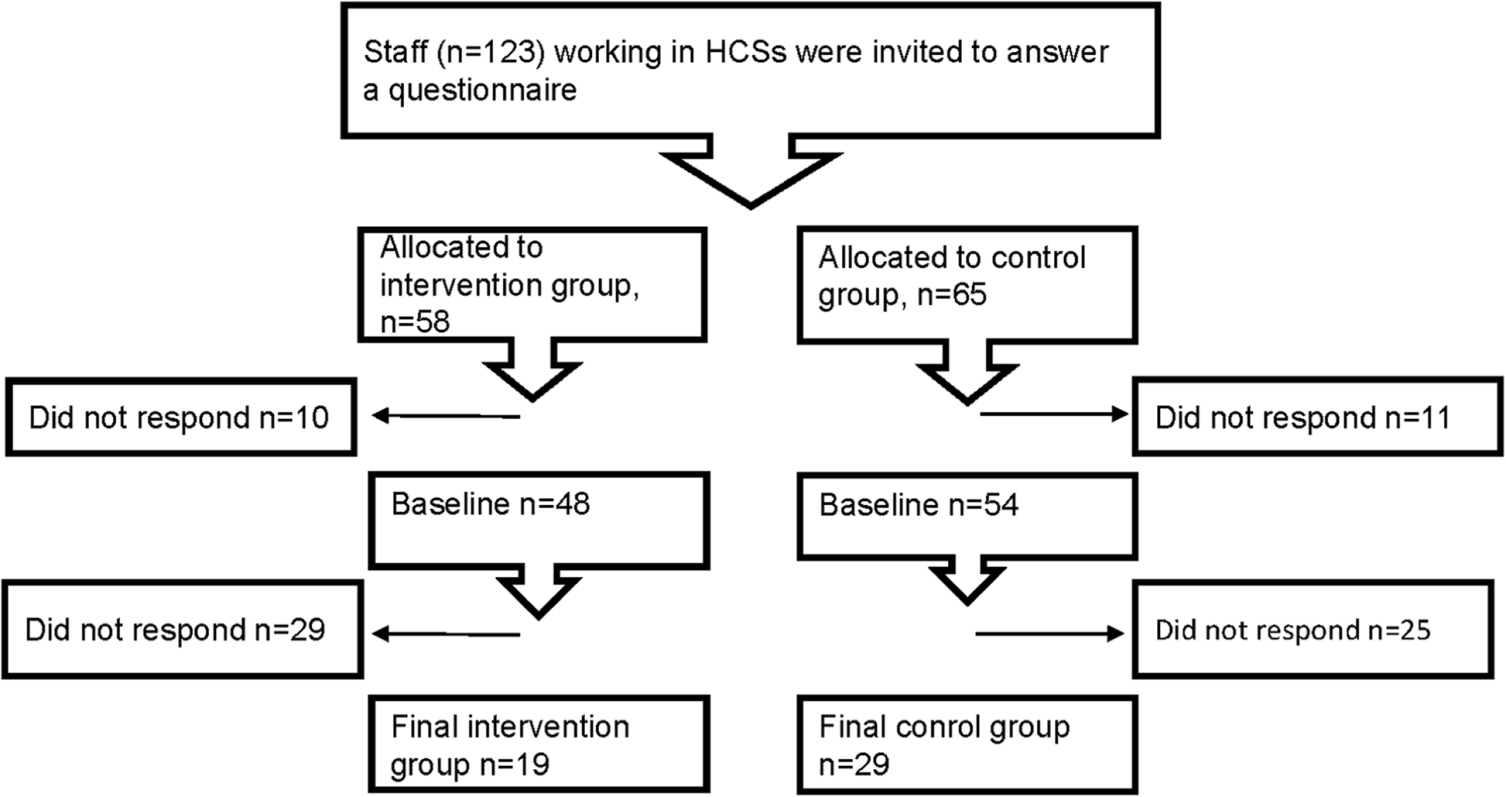
Flow chart of home care service (HCS) staff participating in the study

Inclusion criteria for staff were: being an enrolled nurse or assistant in the HCS, being employed in one of the five HCS districts, and speaking and understanding Swedish. Exclusion criteria were: having been employed by HCS for < 1 year. Of 123 staff invited to take part, 102 (83%) agreed to participate at baseline, and 48 (39%) were included in the study (Fig. 2).

### Intervention

To increase the HCS recipients’ influence on their own care, a PCC intervention was implemented. This intervention was based on the theoretical concepts of person-centredness, using the definition by Ekman et al. [16] which is based on the theory by McCormack & McCance [18]. According to this, there should be a partnership between the care staff and the person in need of care, based on the person’s preferences, shared decision making, documentation [16] and health promotion [31].

#### Educational programme

A Web-based educational programme was developed to be available to staff as support. Because the staff had varied experiences of using a Web-based platform, the education was also given in face-to-face seminars. The aim of the educational programme was to give staff tools to gather information about needs that would increase HCS recipients’ wellbeing. In a conversation with the HCS recipient, staff need to be able to gather information about preferences and, through shared decision making, document a care plan.

The education was given by the researchers (K.L., K.B., P.O.S.) in municipality buildings in the respective districts. After a kick-off including information and an introduction, the first seminar lasting 90 min consisted of oral presentations and discussions on the main theoretical components of PCC, and health-promoting conversations [32]. A second seminar lasting 180 min included supervised skills training in PCC and a health-promoting conversation. The conversation aimed to explore the care-related needs and wishes of a person receiving care from the HCS and began with the researchers asking the participants what would add significant value to their wellbeing. In this way, participants were encouraged to identify care interventions that could promote health. The training was based on Kolb’s experimental learning model [33] including a circular movement between experiences of health-promoting conversations, and reflection. Participants were divided into small groups and in the first exercise they practised using role-play to identify older adults’ specific needs. The second exercise involved role-play in pairs; the goal was to formulate a change plan aimed to increase PCC in HCS. To further develop their skills, the staff were engaged in discussions interspersed with reflective questions such as: What happened in the conversation? What does it mean? What can I learn from this? How can I use what I have learned?

#### Operationalization

After the education, the staff were instructed to operationalize the conversation with the HCS recipient participating in the intervention group. The purpose of the conversation was to explore whether the earlier care plan met the HCS recipient’s expressed need, and if not, to change the care plan in order to maximize health and satisfy psychosocial as well as physical needs. The staff were also encouraged to ensure that the PCC was flexible on a daily basis. This was to enable them to be flexible if a care recipient asked to prioritize tasks differently from the planned or change tasks, provided of course their work schedule allowed for these changes.

During the implementation phase, the staff were invited to take part in two supervisory sessions aimed to support and facilitate the implementation. The control group received and provided care as usual.

### Data collection

#### Baseline

The staff were asked to give a sealed A4 envelope to the HCS recipient. The envelope contained information about the study, the survey and a prepaid envelope for answers. After 2 weeks, the remaining envelopes that had not been distributed by staff because of a high workload, were sent by mail to the HCS recipients. Staff received information about the study at a workplace meeting and were asked to answer the survey.

#### Follow-up

All participants who had answered the baseline survey received a follow-up survey after implementation of the educational programme. Because of organizational difficulties to conduct the education programme according to the timeline in the study protocol [30], the intervention period was prolonged and follow-up was done 20 months after baseline.

### Survey

#### Home care recipients’ perspectives

Health-related quality of life as the primary outcome was assessed using the EuroQol-five dimensions’ scale, five-level version (EQ-5D 5 L), and the NHP. The EQ-5D 5 L consists of two parts. Part one is a state of health description including five dimensions: mobility; self-care; usual activities; pain/discomfort; and anxiety/depression. Each dimension is scored on a 5-point Likert scale ranging from “No complaints” = 0, to “Extreme complaints” = 4. The answers were then transformed using a tariff value which is based on preferences originally derived from a British general population [34]. The tariff value ranges from − 0.53 (“Worse than death”) to 1 (“Full health”), anchoring “Dead” at 0. Part two is a visual analogue scale (VAS) where participants rate overall health between the endpoints “Worst imaginable health” = 0, and “Best imaginable health” = 100. The EQ-5D 5 L has been found to have face and content validity [35]. In this study, Cronbach’s alpha was 0.74.

The NHP includes 38 items and consists of six dimensions: energy level; pain; emotional reaction; sleep; social isolation; and physical abilities. Each item in the NHP is answered by “Yes” or “No” and the score ranges from “Best possible” = 0, to “Worst possible” = 100 [36]. The reliability and validity of assessing HRQoL have been found to be good [37]. In this study, Cronbach’s alpha for the NHP varied between 0.53 and 0.79.

Two secondary outcomes were explored: thriving, and impact on self-determination among HCS recipients. Thriving was measured using the Thriving of Older People Assessment Scale (TOPAS) [38]. The TOPAS includes 32 items and consists of five subscales: residents’ attitude towards the place they are currently living in; quality of care and caregivers; HCS recipients’ activities and peer relationships; opportunities to keep in touch with people and places of importance; and quality of the physical environment. Each item is rated on a 6-point Likert-scale, ranging from “No” = 1, to “Yes, I agree completely” = 6. The scale has been found to be valid and the reliability, using Cronbach’s alpha, of the entire scale has been reported to be 0.95 [39]. The five subscales have shown an internal consistency of 0.83–0.95 when assessing thriving among nursing home residents. In this study, Cronbach’s alpha ranged from 0.85 to 0.94.

Self-determination was assessed using the Impact on Participation and Autonomy–Older Persons (IPA-O) questionnaire [40]. The original scale includes 22 items and consists of six dimensions; however, for reasons of relevance and in view of the aim of this study, four dimensions of self-determination were used, namely: mobility; self-care; activities in and around the house; and having social relationships. Regarding face validity, the questions were assessed to be relevant and important. Hammar et al. [40] have confirmed the IPA-O’s face and content validity. In this study, Cronbach’s alpha ranged from 0.74 to 0.86.

#### Staff perspectives

To assess outcomes among staff, three instruments were used: the Measure of Job Satisfaction scale [41], the Person-Centred Care Assessment Tool (P-CAT) [42], and the Stress of Conscience scale [43].

The Measure of Job Satisfaction scale includes 37 items and consists of five dimensions: personal satisfaction; satisfaction with workload; team spirit; training; and professional support. Responses are scored on a 5-point Likert scale, ranging from “Very dissatisfied” = 1, to “Very satisfied” = 5. The scale has been found to be valid and reliable [41]. In this study, Cronbach’s alpha was 0.935.

The P-CAT consists of 13 items concerning the content of care, and the environment and work organization. In the present study the P-CAT was used to evaluate changes in PCC. Response alternatives in the P-CAT are scored on a 5-point Likert scale, ranging from “Disagree completely” = 1, to “Agree completely” = 5. Higher scores indicate a greater degree of PCC. The P-CAT has been found to be both valid and reliable [42]. In this study, Cronbach’s alpha was 0.705.

The Stress of Conscience scale consists of ten items related to various health care situations. Each question comprises two parts, A and B. The response alternatives in part A are scored on a 6-point Likert scale, ranging from “Never” = 0, to “Every day” = 5. The questions are related to how often different situations arise in the workplace. Part B comprises a 10 cm VAS on which the participant scores the impact of each situation on their conscience. A total index can be calculated, where a higher value signifies higher levels of stress of conscience. The Stress of Conscience scale has been found to be valid [43]. In this study, Cronbach’s alpha was 0.876.

### Data analysis

To analyse participant characteristics, descriptive analysis was used. Categorical variables are presented as numbers and percentages, and continuous variables are presented as means and standard deviations (SDs), or median and quartiles, depending on whether the data was sufficiently normally distributed. The Results section presents differences in changes with within- and between-group analyses. The ratings were somewhat skewed in several scales in both the HCS recipient group and the staff group.

#### Home care recipients

Because the groups sizes were large enough for the HCS recipient group, parametric analyses were used. Differences within groups were analysed with paired-sample *t*-test and differences in changes between groups were analysed with linear regression (with change as outcome) and adjusting for variables with significant differences in background characteristics between intervention and control group. Effect sizes were analysed using Cohen’s *d*.

#### Staff

Due to the fact that there were insufficient numbers of staff in the study (*n* = < 30), analyses among staff was performed with non-parametric analyses; differences within groups were analysed with the Wilcoxon signed-rank test and differences in changes between groups were analysed using the Mann-Whitney U-test. Due to the non-parametric analyses and small sample size, no adjusting variables were included in the procedure.

Missing values below 10% were replaced with individual mean values for the subscale; where more than 10% of data were missing, the case was excluded [cf. [44]]. This means that *n* varies in the presentation of the outcomes (Tables 3 and 4). A *p*-value of < 0.05 was considered statistically significant in all analyses. Two items in one of the TOPAS subscales were accidentally omitted in the printing of the baseline survey documents. In the analysis the same items in the follow-up survey were omitted and the results are therefore based on 30 items. A statistical test of the effect of omitting the two items suggests that the impact on the estimation of the concept was limited [11]. Statistical calculations were performed using IBM SPSS Statistics version 25.0 (IBM Corp., Armonk, NY, USA).

## Results

The included HCS recipients were between 65 and 100 years old. Most lived alone and in apartments, and more than half of them received visits from HCS staff once a day or more. Significantly more participants in the control group compared with the intervention group lived alone in apartments (Table 1).

**Table 1:** HCS recipients’ background characteristics, analysed using descriptive analysis

Background characteristic	Intervention group (*n* = 40)	Control group (*n* = 41)	*P*-value
Sex			
Female, n (%)	25 (62)	30 (73)	0.347
Mean age, yrs. (SD, range)	83 (65–98)	84 (65–100)	0.684
Living alone, n (%)	29 (72)	37 (90)	0.049*
Housing, n (%)			
Apartment	27 (71)	38 (95)	0.012*
House	11 (29)	3 (5)	
Education, n (%)			
Primary school	16 (43)	21 (52)	0.656
Secondary school	13 (35)	13 (32)	
University	8 (22)	6 (15)	
Country/region of birth, n (%)			
Sweden	37 (95)	34 (85)	0.345
Scandinavia	2 (5)	5 (13)	
Other	0	1 (2)	
Visits from the HCS, n (%)			
Once or several times/day	21 (55)	25 (61)	0.317
1–6 times/week	9 (24)	13 (32)	
Once every second week	7 (18)	3 (7)	

*p-*value = differences between background characteristics of the intervention group compared with the control group. Internal missing values were not included in the analysis

*HCS* Home care service

*SD* Standard deviation

* = statistical significant result

The included HCS staff were between 22 and 63 years old, with a mean age of 42 years. There were no significant differences between the intervention group and the control group with respect to background characteristics except for employment type, where significantly more staff in the control group had permanent employment (Table 2).

**Table 2:** Staff background characteristics, analysed using descriptive analysis

Background characteristic	Intervention group (*n* = 19)	Control group (*n* = 29)	*P*-value
Sex			0.118
Male, n (%)	8 (42)	5 (17)	
Mean age, years (SD, range)	39 (11, 24–58)	43 (13, 22–63)	
Contact with the older persons, n (%)			0.110
Daily	4 (22)	9 (31)	
Once a week	9 (50)	19 (66)	
Every month or less often	5 (28)	1 (3)	
Frequency of contact with the older persons’ family members, n (%)			0.888
Daily	0 (0)	0 (0)	
Once a week	2 (11)	3 (10)	
Every month	10 (52)	14 (48)	
Yearly or less often	7 (37)	12 (42)	
Recipients’ need for nursing care, n (%)			0.204
With almost everything	5 (28)	6 (21)	
With some things	11 (61)	22 (79)	
With almost nothing	2 (11)	0 (0)	
Education level in nursing, n (%)			0.217
No nursing education	6 (33)	4 (14)	
Care assistant	3 (17)	4 (14)	
Enrolled nurse	9 (50)	21 (72)	
Employment type, n (%)			**0.011**
Permanent	14 (74)	28 (97)	
Temporary	5 (26)	1 (3)	

*p*-value = differences between background characteristics of the intervention group compared with the control group. Internal missing values were not included in the analysis

*SD* Standard deviation

The results are presented from the perspective of the HCS recipients (Table 3) and staff (Table 4). Overall, we found statistically significant differences between HCS recipients’ before and after ratings of thriving and HRQoL but no significant effect on self-reported self-determination. The effect size, calculated with Cohen’s *d*, was small in the within-group analysis and small to moderate in the between-group analysis. Among staff, no significant effects of the intervention were found.

**Table 3:** Within and between-group analysis of health-related quality of life (HRQoL), thriving and self-determination among home care receivers

	Within group analysis Intervention group^**a**^				Cohens’ d	Within group analysis Control group^a^				Cohens’ d	Between group analysis Intervention/Contol ^**b**^		Cohens d
	n	Baseline Mean (SD)	Follow-up Mean (SD)	*p*-value		n	Baseline Mean (SD)	Follow-up Mean (SD)	*p*-value		*p*-value		
EQ-5D (−0.53–1.0)	27	0.68(0.2)	0.66(0.18)	0.616		28	0.71 (0.24)	0.64 (25)	0.051		0.288	(−0.148, 0.450)	
EQ-VAS (0–100)	33	52 (19)	53(22)	0.756		34	62 (22)	56 (23)	0.045*	0.27	0.196	−16.525, 3.456	
NHP (0–100)													
Energy	31	43 (39)	54 (41)	0.025*	0.27	31	43 (39)	51 (44)	0.198		0.516	−21.682, 11.016	
Pain	29	33 (32)	31 (32)	0.614		27	31 (33)	31 (31)	0.885		0.571	−6.989, 12.525	
Emotions	29	17 (17)	24 (23)	0.039*	0.35	26	14 (18)	14 (23)	0.862		**0.048**	**(−17.435, −0.098)**	**0,43**
Sleep	34	36 (30)	37 (27)	0.872		29	31 (24)	29 (26)	0.515		0.677	−14.558, 9.526	
Isolation	34	16 (19)	18 (19)	0.521		30	13 (16)	18 (22)	0.229		0.331	−5.051,14.761	
Mobility	30	44 (24)	46 (24)	0.682		28	46 (30)	50 (34)	0.271		0.708	−8.482,12.398	
TOPAS (number of scores in scale)													
Total score (32–192)	19	157 (17)	153 (17)	0.354		17	151 (18)	144 (22)	0.009*	0.35	0.325	−16.147, 5.514	
Residents’ attitude (4–24)	35	23 (2)	22 (3)	0.110		34	23 (2)	22 (2)	0.472		0.306	−0.564, 1.769	
Quality of care (9–54)	33	50 (4)	49 (4)	0.540		29	49 (4)	48 (6)	0.026*	0.20	0.159	−4.448, 0.746	
Activities and peer relationships (8–48)	23	33 (12)	34 (11)	0.531		21	31 (11)	27 (12)	0.015*		0.026*	**(−10. 766, −0.717)**	**0,75**
Opportunities to keep in touch (4–24)	32	19 (4)	18 (5)	0.147		29	19 (5)	18 (5)	0.114		0.820	−2.076, 2.609	
Physical environment (5–25)	32	29 (2)	28 (2)	0.273		29	28 (2)	28 (3)	0.019*	0.0	0.715	−1.168, 0.806	
IPA-O (number of scores in scale)													
Self-determination, mobility (4–20)	36	6.2 (3.1)	6.7 (3.8)	0.505		35	6.3 (4.0)	6.0 (2.9)	0.623		0.694	−2.590, 1.734	
Self-determination, self-care (5–25)	36	5.6 (1.4)	6.0 (2.3)	0.180		37	6.5 (4.1)	6.7 (2.8)	0.757		0.771	−1.718, 1.279	
Self-determination, household activities (4–20)	25	7.0 (4.3)	8.1 (3.8)	0.202		27	9.1 (3.5)	7.7 (3.9)	0.099		0.060	−5.174, 0.166	
Social relationships (5–25)	34	7.5 (2.6)	8.4 (2.9)	0.041*	0.33	37	8.3 (4.0)	8.4 (3.8)	0.877		0.261	−2.296, 0.633	

^a^Paired-sample *t*-test

^b^ between group analysis of within group changes using linear regression adjusting for the variables Living alone and Housing

*Statistically significant difference *p*-value ≥0.05

*EQ-5D* EuroQol-five dimensions, *EQ-VAS* = EuroQol visual analogue scale, *IPA-O* Impact on Participation and Autonomy – Older Persons questionnaire, *NHP* Nottingham Health Profile, *SD* Standard deviation, *TOPAS* Thriving of Older People Assessment Scale

For the EQ-5D, EQ-VAS, TOPAS, a high value = positive outcome; for NHP and IPA-O, a high value = negative outcome

**Table 4:** Within and between-group analysis in self-reported job satisfaction, person-centred care (PCC) and stress of conscience among staff

	Intervention group^**a**^				Control group^**b**^				Intervention/ Control^**b**^
	n	Baseline M (Q1; Q3)	Follow-up M (Q1; Q3)	*p*-value	n	Baseline M (Q1; Q3)	Follow-up M (Q1; Q3)	*p*-value	*p*-value
**Measure of job satisfaction** (number of scores in scale)									
Overall job satisfaction (37–185)	17	131 (120; 140)	131 (116; 148)	0.733	22	123 (109; 136)	122 (111; 136)	0.468	0.804
Personal satisfaction (10–50)	18	39 (37; 41)	39 (37; 41)	0.888	27	37 (31; 41)	38 (35; 42)	0.571	0.836
Satisfaction with workload (7–35)	17	26 (20; 27)	25 (19; 28)	0.697	27	21 (17; 24)	21 (18; 24)	0.705	0.972
Satisfaction with professional support (9–45)	18	34 (32; 38)	34 (31; 36)	0.795	28	34 (30; 38)	33 (31; 37)	0.980	0.892
Satisfaction with pay (7–35)	18	20 (17; 24)	21 (16; 26)	0.864	25	21 (17; 24)	22 (17; 25)	0.955	0.881
Satisfaction with training (4–20)	18	13 (12; 15)	13 (10; 16)	0.958	27	13 (10; 13)	12 (9; 15)	0.730	0.726
Overall person-centred care (13–65)	18	44 (41; 49)	43 (39; 52)	0.756	25	45 (39; 51)	46 (41; 54)	0.432	0.937
Personalizing care (7–35)	18	27 (24; 28)	27 (22; 31)	0.448	28	26 (23; 30)	26 (26; 29	0.853	0.306
Organizational support (4–20)	18	12 (10; 16)	13 (10; 15)	0.855	27	13 (11; 15)	14 (11; 17)	0.234	0.308
Environmental accessibility (2–10)	18	6 (6; 7)	6 (6; 7)	0.567	28	6 (5; 7)	7 (6; 8)	0.772	0.867
**Stress of conscience** (number of scores in scale)									
Overall stress of conscience (0–45)	17	7 (1.5; 14)	7 (2.75; 13)	0.123	25	18 (8; 23)	12 (7; 17.5)	0.037*	0.625
How often do you lack time to provide the care the patient needs?	18	2 (0; 3.25)	1 (0; 3)	0.142	27	3 (2; 4)	3 (2; 4)	0.662	0.184
Are you ever forced to provide care that feels wrong?	18	1.5 (0; 3)	1 (0; 2)	0.290	28	1 (0; 3)	1 (0.25; 2)	0.423	0.851
Do you ever have to deal with incompatible demands in your work?	18	1.5 (0; 3)	1 (0; 3)	0.676	27	2 (1; 3)	2 (1; 3)	0.580	0.877
Do you ever see patients being insulted and/or injured?	18	0 (0; 1)	0 (0; 1)	0.619	27	1 (0; 3)	0 (0; 1)	0.015*	0.086
Do you ever find yourself avoiding patients or family members who need help or support?	18	0 (0; 0)	0 (0; 1)	0.417	29	0 (0; 1)	0 (0; 0)	0.117	0.062
Is your private life ever so demanding that you do not have the energy to devote yourself to your work as you would like?	17	0 (0; 0)	0 (0; 1)	1	29	1 (0; 2.5)	0 (0; 2)	0.689	0.571
Is your work in health care ever so demanding that you do not have the energy to devote yourself to your family the way you would like?	18	2 (0; 3)	1 (1; 3)	0.328	29	3 (1; 4)	3 (2; 4)	0.291	0.226
Do you ever feel that you cannot live up to others’ expectations of your work?	17	0.5 (0; 2.25)	0 (0; 1.25)	0.435	28	2 (1; 3)	1 (0.25; 2.75)	0.267	0.922
Do you ever lower your aspirations to provide good care?	17	0 (0; 3)	0.5 (0; 2)	0.596	25	2 (1; 3)	1 (0; 2)	0.057	0.214

^a^Wilcoxon signed-rank test; ^b^Mann-Whitney U-test

*Significant difference *p*-value ≥0.05

*M* Median, *Q1* First quartile, Q3 Third quartile

### Home care recipients

The within-group analysis showed that, at follow-up, the *intervention group* reported decreased HRQoL in respect of energy (*p* = 0.025) and emotions (*p* = 0.039) (NHP). In addition, self-determination, scored on the subscale of social contacts, showed a decrease at follow-up (*p* = 0.041). The other variables had not changed.

The *control group* reported decreased HRQoL (*p* = 0.045) on the EuroQol VAS (EQ-VAS). They also had decreased self-reported HRQoL using the EQ-5D 5 L, but this did not quite reach the level of significance (*p* = 0.051). Furthermore, their TOPAS results showed decreased thriving in the total score (*p* = 0.009) as well as decreased scores for the subscales care quality (*p* = 0.026), activities (*p* = 0.015) and physical environment (*p* = 0.019).

The between-group analysis showed a significant difference in groups regarding the change in thriving, measured using the TOPAS, in the subscale activities and peer relationships (*p* = 0.026). The *intervention group* were relatively stable regarding activities and peer relationships while the control group showed a decrease in activities and peer relationships. There was a significant difference in change in HRQoL regarding the subscale emotions where the intervention group had slightly increase in negative emotions (*p* = 0.048) (Table 3).

### Staff

The between-group analysis showed no significant differences. There were no significant differences within the intervention group. Within-group analysis of the control group showed a significant decrease in self-reported overall stress of conscience (*p* = 0.037) and a decrease in one item of the Stress of Conscience scale regarding patients being insulted and/or injured (*p* = 0.015) (Table 4).

## Discussion

The study aimed to evaluate effects of a person-centred and health-promoting HCS intervention on HRQoL, thriving, and self-determination among older people, and on job satisfaction, stress of conscience, and level of PCC among care staff. The findings showed that the intervention contributed to maintaining high thriving levels; by contrast, a decline was found in the control group of older people. The intervention group also had a slightly increase in negative emotions. No significant effect was found among participating staff. To our knowledge, the present study is one of a very few in-depth, extensive intervention studies on HCSs that include a control group.

Our main finding in the older people was that thriving was relatively stable in the intervention group but that it decreased over time in the control group. However, when comparing within-group differences between the intervention and the control group, only “being engaged in activities” remained significantly different. This dimension included taking part in meaningful, enjoyable activities that matched the older people’s interest. As we reported previously [11], thriving was rated relatively high and the dimension “being engaged in activities” was rated lowest of all dimensions at baseline. The finding that the other dimensions did not improve seems reasonable as a high score at baseline limits the possibility of improvement.

This is the first time that thriving, evaluated using the TOPAS, has been used as outcome variable in an intervention study. The scale has previously been used in cross-sectional studies, with comparable values [11, 12, 45], which suggests that the scale has a ceiling effect and may need to be further developed and tested. Furthermore, the period between baseline and follow-up was 20 months, which probably also had an impact on the results. Considering the age of the population, it is reasonable to believe that health would decline and that the possibility to take part in activities would be reduced. One speculation is that the intervention may have a preventive effect. For instance, the stable effect in the intervention group may have been the result of a special effort made by the staff to bring social support to the members of this group; by contrast, the decline in the control group mirrored a natural process.

Another significant difference between groups, in terms of within-group changes from baseline to follow-up, was a score for emotions as part of the NHP. The HCS recipients in the intervention group reported an increase in negative emotions compared to the control group. Influencing emotions were never hypothesized or targeted in the intervention, and thus this seemingly random finding is difficult to interpret meaningfully. Could it be that the extensive intervention was perceived onerous by participants and thereby influencing negative emotions, or was this a random finding emerging at subscale level of the NHP? Further exploration would be valuable.

Based on the EQ-5D and EQ-VAS scores, the HCS recipients in the control group experienced a decrease in HRQoL from baseline to follow-up while the intervention group did not report any change. A comparison of within-group changes showed no significant difference. Reporting on patients with heart failure in palliative HC, Brännström & Boman [46] showed that their intervention group had a small but significant increase in HRQoL compared with usual care. Theirs was a between-group analysis assessed using the EQ-VAS but not the EQ-5D. Possibly an important difference between their study and ours is the number of staff included in the interventions (*n* = 7 vs *n* = 48) as it may be easier to have control over and give support to a small number of staff, as in Brännström & Boman’s study [38]. To illustrate, Ekman et al. [47], in their study of PCC in one setting in a hospitals. The study was conducted during 2008–2010 and number of staff is not presented, it seems, however, reasonable to believe that a relatively large number of staff had been involved in care. The authors [47] found that not all staff provided PCC as intended. This suggests that the number of staff may be crucial, a small staff group may be easier to support when introducing a new care model. Further, the older persons participating in the study by Ekman et al. [47] received care from staff who had received PCC education. A weakness in our study is that care was also given by temporary staff who had not received PCC education, which may have had an impact on the care given and therefore the results of the study.

Studies about PCC have also been conducted in the context of hospitals. In a study by Olsson et al. [48], patients with total hip arthroplasty receiving PCC in hospital did not rate higher HRQoL; in other words, the intervention group and control group had a comparable increase in HRQoL. In Hansson et al’s hospital study of PCC among patients with chronic heart failure [49], HRQoL was slightly higher in the intervention group compared with the group receiving conventional care, but did not reach a level of significance. The authors suggested that their study was underpowered, which is probably the case also in our study.

Among the staff in our study, the control group reported a lower level of self-reported stress of conscience at follow-up compared with baseline. This difference can be explained by that a low number of participants makes it possible for specific events to have great influence on the results. For example, at baseline, some staff reported a higher level of stress related to having observed how older adults were mistreated, i.e., insulted and/or injured. The change at follow-up may therefore be due to changes in the staff group or HCS recipient group, which can, depending on the size of the study group, have a great impact.

A lack of results among staff, similar to our findings, has been reported in a previous systematic review about the impact of PCC interventions on staff working behaviours in the context of dementia care [24]. The results were varied and showed that only two out of eleven studies reported a high rating on staff working behaviours. One of the studies by Burgio et al. [50] reported a significant improvement in communication skills in the intervention group. By contrast, Sprangers et al. [51] did not find any changes in their intervention group compared with their control group.

As in our study, Bökberg et al. [52], who evaluated an educational intervention concerning person-centred palliative care, found no significant changes in respect of PCC in nursing homes. They concluded that the level of PCC self-reported by staff was already high at baseline, which allowed only few possibilities for improvement in a follow-up after the intervention.

A limitation in the study is the large number of tests performed that will lead to a higher risk of type 1 errors. This is important to consider in combination with the limited significant results and the findings are best seen as tentative, needing further study and thus interpretated with caution.

The sample size of HCS recipients in our study was small for several reasons. It is possible that the HCS recipients were satisfied with the HCS and consequently lacked motivation to participate. Satisfaction with care can be measured using the TOPAS and its subscale of care quality with a maximum possible score of 54. In our population, the mean score in the intervention and the control group varied between 48 and 50. It is noteworthy, however, that the sample had a relatively high mean age and mean age has been found to have a strong association with satisfaction with care [53].

Another possible reason for our difficulties to include more participants is that many HCS recipients were too frail and therefore hesitated to take part in research activities and answer a survey. The included participants, who had a mean age of 84 years, rated their HRQoL relatively high and their EQ-5D scores varied between 0.64 and 0.71. It is possible that many older persons in need of HCS have lower HRQoL than our sample and that such persons may have declined to participate.

One reason for the high dropout rate may be that the intervention had a prolonged implementation period. This can be a problem especially in HCS contexts, as HCS recipients are often of high age, with co-morbidities and frailty. Deterioration in health over time can reasonably be expected, and may result in the need to change housing or hospitalization. Also from the point of view of staff, the intervention length was challenging in light of high staff turnover rates. The plan was to have an intensive education period where staff could take part in a Web-based educational programme. However, the HCS leaders did not agree to the plan and wanted to gather the staff in group meetings instead. Because of the heavy workload of, and sick leave among, staff, as well as organizational changes and a change of leaders, there were extensive difficulties to arrange the planned group meetings, and meetings had to be postponed several times. Hence, the timeline was inevitably extended and an education period that was planned to take 2 months using a Web-based approach took 1 year and 2 months to complete. Besides the risk of having dropouts among HCS recipients during this prolonged period, it is also likely that it was difficult for staff participants to keep focus on the intervention as the interval between intervention meetings became lengthy. In view of the prolonged period, a booster education would probably have been suitable; however, in this case, too, the onerous clinical workload of participating staff was a hindering factor. To implement complex interventions in organizations undergoing austerity and rationalization is highly demanding and needs careful thought and strong mutual ownership to maintain fidelity to the intervention.

## Conclusions

The intervention contributed to maintaining high levels of thriving in contrast to low levels found in the control group, and it seems reasonable to consider the intervention focus on staff as more person-centred and health-promoting. The finding that the intervention group had increase in negative emotions is difficult to interpret, and warrants further exploration. The lack of post-intervention changes in staff may relate to limited sample power as well as limited instrumentation sensitivity. Even though the results are sparse, the challenges discussed may be of importance for future studies in the context of HCS.

## Data Availability

The data sets used and/or analysed during the current study are available from the corresponding author on reasonable request.

## Abbreviations

EQ-5D: EuroQol-five dimensions
EQ-5D 5 L: EuroQol-five dimensions, five-level version
EQ-VAS: EuroQol visual analogue scale
HC: Home care
HCS: Home care service
HRQoL: Health-related quality of life
IPA-O: Impact on Participation and Autonomy–Older Persons
P-CAT: Person-Centred Care Assessment Tool
PCC: Person-centred care
QoL: Quality of life
SD: Standard deviation
TOPAS: Thriving of Older People Assessment Scale
VAS: Visual analogue scale

## References

[1] Grabowski DC (2006). The cost-effectiveness of noninstitutional long-term care services: review and synthesis of the most recent evidence. Med Care Res Rev.

[2] Kaye HS, LaPlante MP, Harrington C (2009). Do noninstitutional long-term care services reduce Medicaid spending?. Health Aff (Millwood).

[3] Harrington C, Jacobsen FF, Panos J, Pollock A, Sutaria S, Szebehely M (2017). Marketization in long-term care: a cross-country comparison of large for-profit nursing home chains. Health Serv Insights.

[4] Newcomer RJ, Ko M, Kang T, Harrington C, Hulett D, Bindman AB (2016). Health care expenditures after initiating long-term services and supports in the community versus in a nursing facility. Med Care.

[5] Wiles JL, Leibing A, Guberman N, Reeve J, Allen RE (2012). The meaning of “aging in place” to older people. Gerontologist.

[6] Ryan AA, McCann S, McKenna H (2009). Impact of community care in enabling older people with complex needs to remain at home. Int J Older People Nursing.

[7] Ryan RM, Deci EL (2000). Self-determination theory and the facilitation of intrinsic motivation, social development, and well-being. Am Psychol.

[8] Jarling A, Rydström I, Ernsth-Bravell M, Nyström M, Dalheim-Englund AC (2018). Becoming a guest in your own home: home care in Sweden from the perspective of older people with multimorbidities. Int J Older People Nursing.

[9] Bölenius K, Lämås K, Sandman P-O, Lindkvist M, Edvardsson D (2019). Perceptions of self-determination and quality of life among Swedish home care recipients-a cross-sectional study. BMC Geriatr.

[10] Haight BK, Barba BE, Tesh AS, Courts NF (2002). Thriving a life span theory. J Gerontol Nurs.

[11] Lämås K, Bölenius K, Sandman PO, Bergland Å, Lindkvist M, Edvardsson D (2020). Thriving among older people living at home with home care services—a cross-sectional study. J Adv Nurs.

[12] Björk S, Lindkvist M, Wimo A, Juthberg C, Bergland A, Edvardsson D. Residents’ engagement in everyday activities and its association with thriving in nursing homes. J Adv Nurs. 2017;73(8):1884–95. 10.1111/jan.13275 28229474

[13] Olaison A. Negotiating needs: processing older persons as home care recipients in gerontological social work practices. Linköping: Linköping University Electronic Press; 2009.

[14] Miranda-Castillo C, Woods B, Orrell M (2010). People with dementia living alone: what are their needs and what kind of support are they receiving?. Int Psychogeriatr.

[15] Sandberg L, Nilsson I, Rosenberg L, Borell L, Boström AM (2019). Home care services for older clients with and without cognitive impairment in Sweden. Health Soc Care Commun.

[16] Ekman I, Swedberg K, Taft C, Lindseth A, Norberg A, Brink E, Carlsson J, Dahlin-Ivanoff S, Johansson I-L, Kjellgren K (2011). Person-centered care—ready for prime time. Eur J Cardiovasc Nurs.

[17] Olsson LE, Jakobsson Ung E, Swedberg K, Ekman I (2013). Efficacy of person-centred care as an intervention in controlled trials–a systematic review. J Clin Nurs.

[18] McCormack B, McCance T (2010). Person-centred nursing : theory and practice.

[19] Sjögren K, Lindkvist M, Sandman PO, Zingmark K, Edvardsson D (2013). Person-centredness and its association with resident well-being in dementia care units. J Adv Nurs.

[20] Holliday RC, Cano S, Freeman JA, Playford ED (2007). Should patients participate in clinical decision making? An optimised balance block design controlled study of goal setting in a rehabilitation unit. J Neurol Neurosurg Psychiatry.

[21] Edvardsson D, Fetherstonhaugh D, McAuliffe L, Nay R, Chenco C (2011). Job satisfaction amongst aged care staff: exploring the influence of person-centered care provision. Int Psychogeriatr.

[22] van den Pol-Grevelink A, Jukema JS, Smits CH (2012). Person-centred care and job satisfaction of caregivers in nursing homes: a systematic review of the impact of different forms of person-centred care on various dimensions of job satisfaction. Int J Geriatr Psychiatry.

[23] Sjögren K, Lindkvist M, Sandman PO, Zingmark K, Edvardsson D (2015). To what extent is the work environment of staff related to person-centred care? A cross-sectional study of residential aged care. J Clin Nurs.

[24] Blake D, Berry K, Brown LJE (2020). A systematic review of the impact of person-centred care interventions on the behaviour of staff working in dementia care. J Adv Nurs.

[25] Fitzsimmons S, Buettner LL (2002). Therapeutic recreation interventions for need-driven dementia-compromised. Am J Alzheimer's Dis Other Dement®.

[26] Eloniemi-Sulkava U, Saarenheimo M, Laakkonen ML, Pietilä M, Savikko N, Kautiainen H, Tilvis RS, Pitkälä KH (2009). Family care as collaboration: effectiveness of a multicomponent support program for elderly couples with dementia. Randomized controlled intervention study. J Am Geriatr Soc.

[27] Egan M, Kessler D, Laporte L, Metcalfe V, Carter M (2007). A pilot randomized controlled trial of community-based occupational therapy in late stroke rehabilitation. Top Stroke Rehabil.

[28] Berglund M, Gillsjö C, Svanström R (2019). Keys to person-centred care to persons living with dementia–experiences from an educational program in Sweden. Dementia.

[29] Meagher G, Szebehely M, Mears J (2016). How institutions matter for job characteristics, quality and experiences: a comparison of home care work for older people in Australia and Sweden. Work Employ Soc.

[30] Bölenius K, Lämås K, Sandman P-O, Edvardsson D (2017). Effects and meanings of a person-centred and health-promoting intervention in home care services-a study protocol of a non-randomised controlled trial. BMC Geriatr.

[31] Milestones in Health Promotion -Statements from Global Conferences http://www.who.int/healthpromotion/Milestones_Health_Promotion_05022010.pdf.

[32] Farbring CÅ (2014). Handbook in motivational interviewing. (in Swedish: Handbok i motiverande samtal - MI : teori, praktik och implementering: samtalsguider, övningar, coachingprotokoll).

[33] Kolb DA (1984). Experimental learning: experience as the source of learning and development.

[34] Dolan P, Gudex C, Kind P, Williams A. A social tariff for EuroQol: results from a UK general population survey. York: University of York: Centre for Health Economics; 1995.

[35] Herdman M, Gudex C, Lloyd A, Janssen M, Kind P, Parkin D, Bonsel G, Badia X (2011). Development and preliminary testing of the new five-level version of EQ-5D (EQ-5D-5L). Qual Life Res.

[36] Hunt SM, McKenna S, McEwen J, Backett E, Williams J, Papp E (1980). A quantitative approach to perceived health status: a validation study. J Epidemiol Community Health.

[37] Baro E, Ferrer M, Vazquez O, Miralles R, Pont A, Esperanza A, Cervera AM, Alonso J (2006). Using the Nottingham health profile (NHP) among older adult inpatients with varying cognitive function. Qual Life Res.

[38] Bergland A, Kirkevold M, Sandman PO, Hofoss D, Vassbo T, Edvardsson D (2014). Thriving in long-term care facilities: instrument development, correspondence between proxy and residents’ self-ratings and internal consistency in the Norwegian version. J Adv Nurs.

[39] Bergland A, Kirkevold M, Sandman PO, Hofoss D, Edvardsson D (2015). The thriving of older people assessment scale: validity and reliability assessments. J Adv Nurs.

[40] Hammar IO, Ekelund C, Wilhelmson K, Eklund K. Impact on participation and autonomy: test of validity and reliability for older persons. Health Psychol Res. 2014;2(3):68–73. 10.4081/hpr.2014.1825 PMC4768594 26973949

[41] Traynor M, Wade B (1993). The development of a measure of job satisfaction for use in monitoring the morale of community nurses in four trusts. J Adv Nurs.

[42] Sjögren K, Lindkvist M, Sandman P-O, Zingmark K, Edvardsson D (2012). Psychometric evaluation of the Swedish version of the person-centered care assessment tool (P-CAT). Int Psychogeriatr.

[43] Glasberg A-L, Eriksson S, Dahlqvist V, Lindahl E, Strandberg G, Söderberg A, Sørlie V, Norberg A (2006). Development and initial validation of the stress of conscience questionnaire. Nurs Ethics.

[44] Shrive FM, Stuart H, Quan H, Ghali WA (2006). Dealing with missing data in a multi-question depression scale: a comparison of imputation methods. BMC Med Res Methodol.

[45] Patomella AH, Sandman PO, Bergland A, Edvardsson D (2016). Characteristics of residents who thrive in nursing home environments: a cross-sectional study. J Adv Nurs.

[46] Brännström M, Boman K (2014). Effects of person-centred and integrated chronic heart failure and palliative home care. PREFER: a randomized controlled study. Eur J Heart Fail.

[47] Ekman I, Wolf A, Olsson L-E, Taft C, Dudas K, Schaufelberger M, Swedberg K (2012). Effects of person-centred care in patients with chronic heart failure: the PCC-HF study. Eur Heart J.

[48] Olsson LE, Karlsson J, Berg U, Kärrholm J, Hansson E (2014). Person-centred care compared with standardized care for patients undergoing total hip arthroplasty—a quasi-experimental study. J Orthop Surg Res.

[49] Hansson E, Ekman I, Swedberg K, Wolf A, Dudas K, Ehlers L, Olsson L-E (2016). Person-centred care for patients with chronic heart failure–a cost–utility analysis. Eur J Cardiovasc Nurs.

[50] Burgio LD, Allen-Burge R, Roth DL, Bourgeois MS, Dijkstra K, Gerstle J, Jackson E, Bankester L (2001). Come talk with me: improving communication between nursing assistants and nursing home residents during care routines. Gerontologist.

[51] Sprangers S, Dijkstra K, Romijn-Luijten A (2015). Communication skills training in a nursing home: effects of a brief intervention on residents and nursing aides. Clin Interv Aging.

[52] Bökberg C, Behm L, Wallerstedt B, Ahlström G (2019). Evaluation of person-centeredness in nursing homes after a palliative care intervention: pre-and post-test experimental design. BMC Palliat Care.

[53] Rahmqvist M, Bara A-C (2010). Patient characteristics and quality dimensions related to patient satisfaction. Int J Qual Health Care.

